# Genetic Structure of the Cave-Dwelling Catfish *Pterocryptis anomala* (Siluriformes: Siluridae) in Southwest China

**DOI:** 10.3390/ani15091202

**Published:** 2025-04-23

**Authors:** Renrong Huang, Jinmei Chen, Hongmei Li, Huan Cheng, Renyi Zhang

**Affiliations:** School of Life Sciences, Guizhou Normal University, Guiyang 550025, China; huang918918@126.com (R.H.); qianfuyu.shen@outlook.com (J.C.); lhm1971374689@163.com (H.L.); 18185299978@163.com (H.C.)

**Keywords:** *Pterocryptis anomala*, Southwest China karst, conservation, genetic differentiation, population structure

## Abstract

The mountainous regions of Southwest China are biodiversity hotspots where geographical isolation promotes genetic differentiation and species diversification. For cave-dwelling species like the *Pterocryptis anomala*, how geographical isolation, historical climate, and riverscapes have influenced their evolution remains largely unexplored. Based on 255 samples from the Pearl River and the Yangtze River, this study integrated two mitochondrial genes and two nuclear genes to analyze the genetic diversity and structure of the *P. anomala* population. Phylogenetic trees based on mitochondrial DNA revealed two distinct clades of *P. anomala*, while nuclear DNA loci showed no clear separation. Spatial Analysis of Molecular Variance (SAMOVA) confirmed two groups: Clade I (the Yangtze, the Guijiang, and the Duliujiang Rivers) and Clade II (the Nanpanjiang, Hongshui, Dahuanjiang, Youjiang, and Rongjiang Rivers). The divergence time between the two clades was estimated at 13.73 million years ago, which was potentially linked to the impact of the QTP uplift on monsoonal systems. The star-like network analysis and neutrality test results indicated that the population of Clade I has maintained a stable state over a long period, while the population of Clade II showed a trend of expansion. Additionally, geographical features such as the Nanling Mountains and the two major river systems may have obstructed gene flow, leading to genetic differentiation. These findings improved our understanding of this species’ evolutionary history and population structure, offering valuable insights for conservation efforts.

## Simple Summary

This study elucidated the genetic structure and diversity of *Pterocryptis anomala* populations in Southwest China based on two mitochondrial and two nuclear genes and further investigated key driving factors influencing their current distribution patterns. Through phylogenetic analysis of 255 individuals, we identified two distinct evolutionary clades. The haplotype network also supported the division of all individuals into two clades. The divergence time between the two clades was estimated at 13.73 million years ago (Mya), which may have been influenced by the impact of the Qinghai–Tibet Plateau (QTP) uplift on monsoonal systems. Our findings enhance the understanding of the phylogeographic history of *P. anomala* in Southwest China and provide a theoretical framework for targeted conservation strategies to safeguard freshwater biodiversity in this ecologically critical region.

## 1. Introduction

Southwest China stands as a global biodiversity hotspot, shaped by its exceptional geological history and climatic complexity [1,2]. This region, a Quaternary glacial refuge [3], occupies a unique biogeographic position at the convergence of Pacific and Indian monsoon systems. Its dramatic elevational gradients—spanning the Qinghai–Tibet Plateau (QTP), the Yunnan–Guizhou Plateau, and the Sichuan Basin—have created three distinct topographic tiers that foster the ecological adaptive differentiation of species. Major mountain systems, including the Himalayas, Kunlun Mountains, and Hengduan Mountains, act as formidable biogeographic barriers, driving species diversification [4]. The region’s hydrologic architecture further enhances its biological significance. Additionally, the region boasts numerous rivers and lakes, with the central and northern parts dominated by rivers in the Yangtze River basin. Meanwhile southern networks converge into the Pearl River Basin—South China’s largest fluvial system comprising the East, West, and North Rivers, along with tributaries like the Beipan and the Liujiang Rivers [5]. These river networks have been profoundly shaped by Neogene uplift events, particularly the formation of the Nanling Mountains, which established genetic barriers between the Yangtze and the Pearl River biotas [6,7,8]. Mao’er Mountain, located in the northeastern part of Guangxi, is the main peak of Yuecheng Ridge—one of the five major ridges of the Nanling Mountains. It served as the source area for several important rivers, including the Lijiang, the Zijiang, and the Xunjiang Rivers, and is also one of the sources of the Liujiang River. Its strategic position bridges the Yangtze River and the Pearl River [9,10]. Furthermore, the uplift of the QTP was closely related to the collision between the Indian Plate and the Asian Plate in the early Cenozoic, which may have been the largest orogenic event in the history of the Earth [11]. This collision not only triggered the significant uplift of the QTP but also profoundly influenced the climate pattern of Asia [12]. The formation of the East Asian monsoon, the South Asian monsoon, and the plateau monsoon, which play a controlling role in the climate of Southwest China, has been closely related to the uplift of the QTP [13]. In addition, the uplift of the QTP also promoted the development of large-scale drainage patterns and provided unique environmental conditions that facilitated speciation and biodiversity of organisms on and around the plateau [11,14]. For example, the species diversity of *Sinocyclocheilus* Fang, 1936 is closely related to the periodic uplift and erosion of the QTP [15]. It can be seen that the formation of the Asian monsoon and its impact on the climate evolution of Southwest China are important manifestations of the uplift of the QTP in terms of climate and biodiversity. This orographic–climatic interplay maintains the humidity gradients and thermal stability essential for preserving paleoendemic lineages in karst ecosystems [16].

The vast karst landscapes in Southwest China, harboring the world’s most extensive cave systems, are one of the typical karst development areas shaped by the uplift of the QTP and the Asian monsoon climate [17,18], sustained unique subterranean ecosystems with diverse troglobitic communities highly sensitive to environmental perturbations [19,20]. These hypogean habitats hosted remarkable endemic radiations, including cave-adapted genera such as *Triplophysa* Rendahl, 1933 [21], *Sinocyclocheilus* [22], and *Protocobitis* Yang & Chen, 1993 [23]. Cavefish (defined as species requiring groundwater environments for critical life-history stages [24,25]) have been categorized into two ecological guilds: typical cavefish exhibiting obligate subterranean adaptations (e.g., anophthalmia and depigmentation) and atypical cavefish lacking such specialization [26]. The diversification of typical cavefish has been influenced by both cave-specific selection pressures and macro-evolutionary drivers such as the Neogene uplift of the QTP. The uplift of the QTP fragmented the ancestral ranges, thus promoting allopatric speciation [27]. Notably, *Sinocyclocheilus* represents one of East Asia’s most spectacular freshwater fish radiations, with over 80 described species exhibiting cave-related morphological innovations [22]. Despite these advances, the evolutionary mechanisms structuring atypical cavefish populations remain poorly resolved. *Pterocryptis anomala* (Herre, 1933), an atypical cave-dwelling catfish co-occurring with obligate subterranean lineages like *Sinocyclocheilus*, has presented an ideal model to disentangle isolation mechanisms in karst ecosystems. This leads us to pose the question: Does its genetic differentiation primarily reflect (1) isolation due to geographical barriers or (2) hydrological segregation across surface river networks? Resolving this dichotomy requires integrative phylogeographic approaches to partition isolation-by-cave versus isolation-by-river effects.

**Figure 1:**
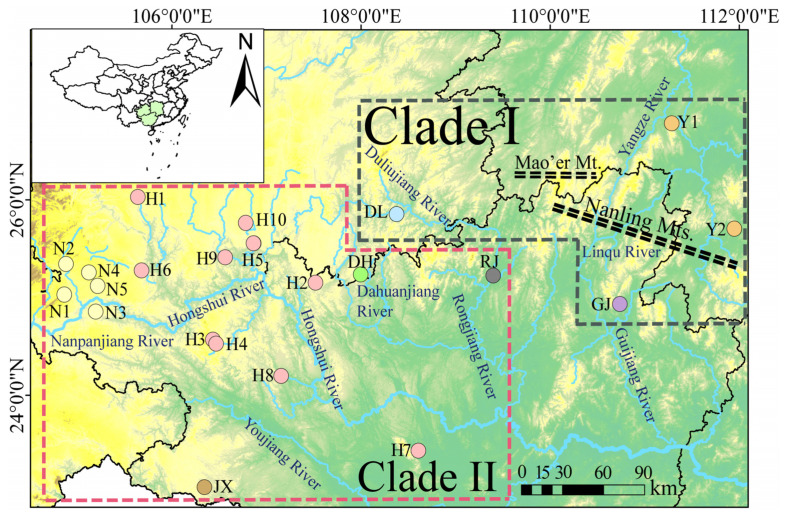
Sampling regions of the *P. anomala* in Southwest China. Different sites were colored according to the tributary. Location codes were consistent with those shown in Table 1.

*Pterocryptis anomala*, a benthic-dwelling catfish endemic to cave and montane stream ecosystems in Southwest China, occupies a unique biogeographic niche spanning the Yangtze River and the Pearl River, including Hunan, Guizhou, and Guangxi provinces (Figure 1) [28]. As a poor disperser with strict habitat fidelity to subterranean and headwater environments [29,30], this species serves as an exceptional model for investigating how historical climatic oscillations, riverine landscape dynamics, and geographic isolation shape freshwater population structure. We employed a multilocus phylogeographic approach, sequencing two mitochondrial genes (the cytochrome c oxidase subunit I, *COI*, and cytochrome *b* gene, Cyt *b*) and two nuclear loci (recombination activating gene 1, *RAG1*, and pleiomorphic adenoma gene-like 2, *PLAGL2*), to analyze genetic structure and differentiation across all populations representing the Yangtze River and the Pearl River. Specific objectives were to (1) delineate spatial genetic architecture across major hydrographic divides and (2) identify drivers (e.g., paleo-drainage rearrangements and karst barriers) underlying contemporary distribution patterns. These analyses will elucidate the phylogeographic history of *P. anomala* populations in Southwest China and establish a theoretical foundation for conservation strategies.

## 2. Materials and Methods

### 2.1. Sample Collection

A total of 22 sites of 255 specimens were collected from both the Yangtze River and the Pearl River for molecular analysis. The geographical distribution of these populations is depicted in Figure 1. Freshly collected specimens from the field were preserved in 75% ethanol and subsequently stored in a −20 °C freezer upon arrival at the laboratory, prior to DNA extraction. All collected specimens are now archived at the College of Life Sciences, Guizhou Normal University, in Guizhou Province, China. The name, location, and sample size of each population are detailed in Table 1.

### 2.2. Laboratory Procedures and Sequence Analyses

Genomic DNA was extracted from *P. anomala* muscle tissues using the traditional high-salt method [31]. We amplified two mitochondrial loci and two nuclear loci via polymerase chain reaction (PCR). Primer sequences are provided in Table S1. Each 35 μL PCR mixture contained 17.5 μL 2× Taq Plus MasterMix (CWBIO, Taizhou, China), 1 μL of each forward/reverse primer (10 μM), 1 μL template DNA (~50 ng/μL), and 14.5 μL ddH₂O. The thermal cycling conditions were as follows: initial denaturation at 95 °C for 5 min, followed by 35 cycles, each cycle consisting of 95 °C for 45 s, annealing at 55−57 °C for 30 s (annealing temperatures: 55 °C for *COI*, Cyt *b*, and *PLAGL2*, 55 °C for *RAG1* in the first stage, and 57 °C for *RAG1* in the second stage), and extension at 72 °C for 1 min, with a final extension at 72 °C for 10 min. For *PLAGL2* and *RAG1*, a nested PCR strategy was implemented, with first-stage products serving as templates for second-stage amplification. In the aforementioned analysis, the nuclear sequences involved are all exon regions. All amplicons were verified by 1.0% agarose gel electrophoresis before bidirectional Sanger sequencing at Sangon Biotech (Shanghai, China) using BigDye Terminator v3.1 chemistry on an ABI 3730xl analyzer.

All sequences were proofread and assembled with the DNA analysis package DNASTAR Lasergene’s SeqMan v7.0 (DNASTAR Inc., Madison, WI, USA). Initially, sequence alignments were conducted utilizing Muscle within MEGA version 11.0 [32]. Subsequently, each protein-coding sequence was translated to verify and designate codon positions. Lastly, the counts of transversions, variable sites, and parsimony-informative sites were determined using MEGA and checked manually. The mitochondrial fragments Cyt *b* and *COI* were concatenated into one mitochondrial locus (mtDNA) for subsequent analyses. Based on mtDNA and nuclear genes, haplotypes were defined using DnaSP v5.0 software [33]. The PHASE algorithm, integrated within the DnaSP, was employed to phase the double chromatograph peaks of nuclear gene sequences, utilizing the default parameter settings. Phasing results were validated based on a posterior probability threshold exceeding 85%, and these validated results were subsequently utilized for further analysis [34]. Sequences of all individuals were deposited in GenBank.

### 2.3. Molecular Phylogenetic Analysis

Utilizing all haplotypes from the concatenated mtDNA sequences (*COI* and Cyt *b*), the phylogenetic relationships among populations of the cavefish *P. anomala* distributed in Southwestern China were reconstructed. This was accomplished through the application of Bayesian Inference (BI) with MrBayes V3.2.1 software [35] and Maximum Likelihood (ML) analysis within the IQ-tree module of PhyloSuite v1.2.2 software [36]. *Clarias fuscus* (Lacepède, 1803), *Ictalurus punctatus* (Rafinesque, 1818), *Ompok bimaculatus* (Bloch, 1794), *Heteropneustes fossilis* (Bloch, 1794), and *Pterocryptis cochinchinensis* (Valenciennes, 1840) were selected as the outgroups for phylogenetic studies. Based on the Akaike Information Criterion (AIC), the optimal nucleotide substitution model, GTR + I + G, was selected for BI analysis using MrModelTest v.2 [37]. The total number of generations for Markov Chain Monte Carlo (MCMC) computation was 2,000,000, with the analysis starting from a random tree and sampling once every 1000 generations. The average standard deviation of split frequencies was less than 0.01, indicating that the sampling of the posterior distribution was sufficient. The ML tree was constructed using IQ-tree module, employing the TVM + F + G model and incorporating the ultrafast bootstrap method. Node support was evaluated through a bootstrap process comprising 5000 replicates. By conducting statistics on the remaining trees, a consensus tree with posterior probability values greater than 50% was generated. Then, the first 25% of the resulting phylogenetic trees was discarded as part of the burn-in process. Finally, the phylogenetic trees were visualized and edited using Interactive Tree of Life (iTOL) v4 [38].

### 2.4. Genetic Diversity and Genetic Differences

Genetic diversity was assessed using DnaSP to calculate haplotype diversity (h), the number of haplotypes (nh), the average number of nucleotide differences (k), the number of variable sites (s), and nucleotide diversity (π). Median-joining networks were constructed using Popart v.1.7 [39] with default settings based on mtDNA and two nuclear genes data to explore the relationships among their respective haplotypes. To assess the degree of genetic differentiation among *P. anomala* from different clades, two statistical methods were employed. Pairwise divergences were calculated both between and within the clades using the Kimura 2-parameter (K2P) model [40] in MEGA. The pairwise fixation index (*F*st) was estimated using Arlequin v.3.5 [41]. To examine the correlation between genetic distance and geographical distance, both matrices were analyzed using Mantel tests in R with the vegan package v2.6-4 [42,43]. The geographical distance matrix was calculated based on the sampling locations (Table S2).

### 2.5. Analysis of Population Structure and Historical Demographic Changes

Based on the aforementioned analyses, all individuals were successfully assigned to corresponding haplotypes. To elucidate the population genetic structure of these haplotypes, we performed Bayesian clustering analysis on the haplotype data using STRUCTURE software v2.3.4 [44]. Initially, the burn-in process was set to 10^5^ steps, followed by 10^6^ Markov chain Monte Carlo (MCMC) iterations. The entire analysis was replicated 10 times for a range of K values from 1 to 10. The optimal number of clusters (K) was determined using STRUCTURE HARVESTER [45] and applying the Delta K method [46]. Subsequently, to consolidate the results from each K replication, CLUMPP v1.1.2 [47] was utilized. Finally, bar plots were generated using DISTRUCT v1.1 [48] to visualize the clustering outcomes. To understand the genetic structure of *P. anomala*, AMOVA analysis was conducted using Arlequin to calculate the distribution of genetic variation within and among populations, as well as the genetic differentiation coefficient between populations. For the AMOVA analysis, *P. anomala* was divided into two geographical groups based on the results of the phylogenetic analysis and possible geographical barriers. The analysis was conducted with 1000 permutations for significance testing. To explore the spatial dynamics of population history and test whether the sequences conform to the expectations of neutrality, Tajima’s *D* [49] and Fu’s *F*s [50] were calculated, and 1000 simulations were performed for each statistic to create a 95% confidence interval. Mismatch distribution analysis was conducted using DnaSP to explore whether past populations had undergone expansion.

### 2.6. Estimations of Divergence Time

Based on the combined mitochondrial gene data, the divergence times among haplotype lineages of all populations were calculated using BEAST v2.4.7 [51], employing the GTR + I + G substitution model. Due to the lack of known fossil records for this group, two calibration points were employed as suggested by Chen et al. [52] and Kappas et al. [53], including (1) the first appearance of the African Clariidae being in the Lower Eocene (34–56 Mya) and (2) the divergence of *Ompok bimaculatus* from *P. cochinchinensis* (22.08 Mya). The outgroups include *Clarisa fuscus*, *Ictalurus punctatus*, *Ompok bimaculatus*, *Heteropneustes fossilis*, and *P. cochinchinensis*. Three separate MCMC chains were run for 20 million generations each, sampling every 1000 generations. Analyses used a relaxed lognormal clock model and a Yule process. Convergence was checked in TRACER v1.7 [54], ensuring effective sample sizes exceeded 200. TreeAnnotator (BEAST v2.4.7 package) was used to generate a maximum clade credibility (MCC) tree, discarding the first 25% as burn-in. The resulting tree, including divergence time estimates, was visualized using FigTree v1.4.3 [55].

## 3. Results

### 3.1. Sequence Information

The 253 sequences were obtained for the mtDNA sequences from all samples of *P. anomala*. The aligned data of the sequences from *P. anomala* had lengths of 651 base pairs (bp) for *COI* and 1138 bp for Cyt *b.* The concatenated mitochondrial genes comprised 1789 bp. There were 31 haplotypes in concatenated mitochondrial gene sequences, among which the frequency of Hap6 was the highest (37.15%), followed by Hap18 (13.83%) and Hap31 (9.49%); Hap6 were shared by two populations (Table S3). The number of polymorphic sites (s) was 89, and the average number of nucleotide differences (k) was 22.284.

The 229 *RAG1* sequences contained 26 polymorphic sites, with an average number of nucleotide differences (k) was 2.716 and the 255 *PLAGL2* sequences contained 157 polymorphic sites and the average number of nucleotide differences (k) was 5.907. For *RAG1*, 30 alleles were identified. Hap1 predominated at a frequency of 40.17%, followed by Hap6 (17.03%) and Hap3 (12.8%). Notably, Hap1 was shared across five populations (Table S4). Similarly, *PLAGL2* revealed 37 alleles, with Hap1 being dominant (53.73%), followed by Hap7 (25.49%) and Hap15 (3.92%). Hap1 exhibited broader distribution, occurring in seven populations (Table S5).

### 3.2. Phylogenetic Analysis and Haplotype Network

**Figure 2:**
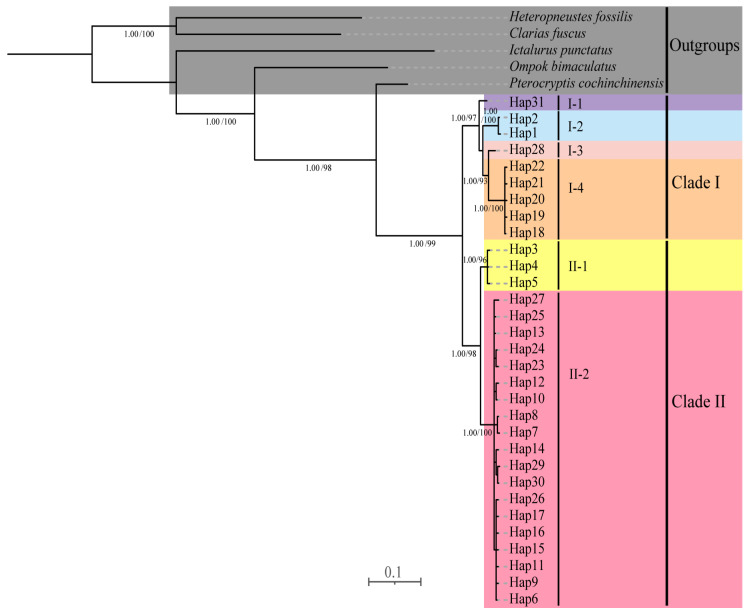
Phylogenetic trees reconstructed based on concatenated mitochondrial gene sequences; nodal values near branches indicate posterior probabilities and bootstrap supports of BI/ML.

**Figure 3:**
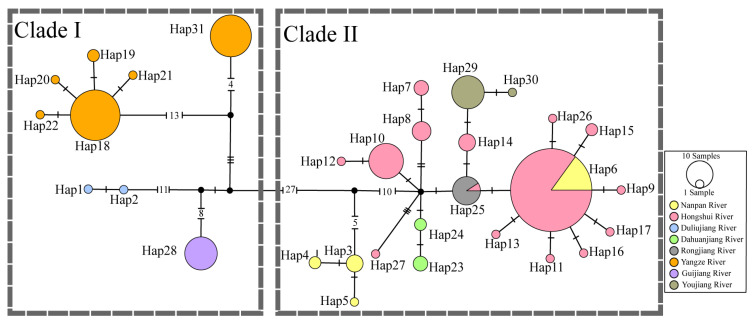
Network profile based on mtDNA data of the populations. Each haplotype is represented by a circle. The size of the circle is proportional to that haplotype’s frequency. Different colors indicate different geographical locations. Location codes are consistent with those shown in Table 1, corresponding to Figure 1.

The phylogenetic trees based on concatenated mitochondrial gene sequences revealed two main branches within the species *P. anomala*; the Clade I consisted of three minor clades: I-1, the sample from DL, belonged to the Duliujiang River; I-2, the sample from GJ, belonged to the Guijiang River; and I-3 and I-4, the samples from Y1 and Y2, which belonged to the Yangtze River. Clade II consisted of two minor clades, II-1 and II-2, which included the remaining *P. anomala* populations, all of which belonged to the Pearl River (Figure 2). Haplotype network analyses revealed distinct genetic structuring, with all studied individuals clustering into two major clades (Figure 3), a pattern that was consistent with both BI and ML phylograms. However, due to the low intraspecific polymorphism and with no significant population differentiation, we did not construct phylogenetic trees based on nuclear genes for the *P. anomala* populations.

The median-joining network based on *RAG1* and *PLAGL2* analysis has a weblike topology, respectively, with many singletons connected through multiple nodes, indicating high genetic variability for *P. anomala* (Figures S1 and S2). In the haplotype networks, some haplotypes were shared between clades, but none had a clear genealogical structure. Three haplotypes based on *RAG1* (Hap1, Hap3, and Hap6) were shared by the two clades (Figure S1). Two haplotypes based on *PLAGL2* (Hap1 and Hap9) were shared by the two clades (Figure S2). Overall, the absence of genetic substructure in nuclear markers was clearly demonstrated in the haplotype network. Specifically, no correlation was detected between haplotype and geographic distribution (Figures S1 and S2).

### 3.3. Genetic Diversity and Population Differentiation

The genetic diversity of *P. anomala* populations in Southwest China is shown in Table 2. Based on mtDNA data, the haplotype diversity (h) of the Duliujiang River site was the highest, with a value of 1.000 ± 0.500, while the Rongjiang River and Guijiang River sites have exhibited the lowest haplotype diversity, both with a value of 0. All other populations had the h value exceeding 0.5, with the exception of the Youjiang River. Regarding nucleotide diversity, the values at different sampling sites ranged from 0.0075 (the Yangtze River site) to 0 (the Rongjiang River and Guijiang River sites). Among them, only two populations (the Yangtze River and Nanpanjiang River sites) had nucleotide diversity (π) values higher than 0.005, while the π values for the remaining six sites fell between 0 and 0.00137 (Table 2). The inter-clade genetic distances (Table 3) ranged from 3.1% (between minor clades I-1 and II-1) to 4% (between minor clades I-4 and II-2).

Based on *RAG1* data, the haplotype diversity (h) of the Rongjiang River site was the highest (0.845 ± 0.039), while the Duliujiang River and Dahuanjiang River sites exhibited the lowest haplotype diversity, both with a value of 0. All other populations had the h value exceeding 0.5, with the exception of the Duliujiang River, Dahuanjiang River, and Hongshui River sites. Regarding nucleotide diversity, the values at different sampling sites ranged from 0.00603 (the Rongjiang River site) to 0 (the Dahuanjiang and Duliujiang Rivers sites). Among them, only the Rongjiang River site had nucleotide diversity (π) values higher than 0.005, while the π values for the remaining seven sites fell between 0 and 0.0045 (Table 2). Based on *PLAGL2* data, the haplotype diversity (h) of the Youjiang River site was the highest (0.874 ± 0.064), while the Duliujiang and Dahuanjiang River sites exhibited the lowest haplotype diversity, both with a value of 0. All other populations had the h value lower than 0.5, with the exception of the Youjiang River and Rongjiang River sites. Regarding nucleotide diversity, the values at different sampling sites ranged from 0.05209 (the Youjiang River site) to 0 (the Dahuanjiang River and Duliujiang River sites). Among them, only the Youjiang River and Nanpanjiang River sites had nucleotide diversity (π) values higher than 0.005, while the π values for the remaining seven sites fell between 0 and 0.00433 (Table 2). Compared to *RAG1* and mtDNA data, the *PLAGL2* gene exhibited a lower level of genetic diversity in all populations (Table 2).

**Figure 4:**
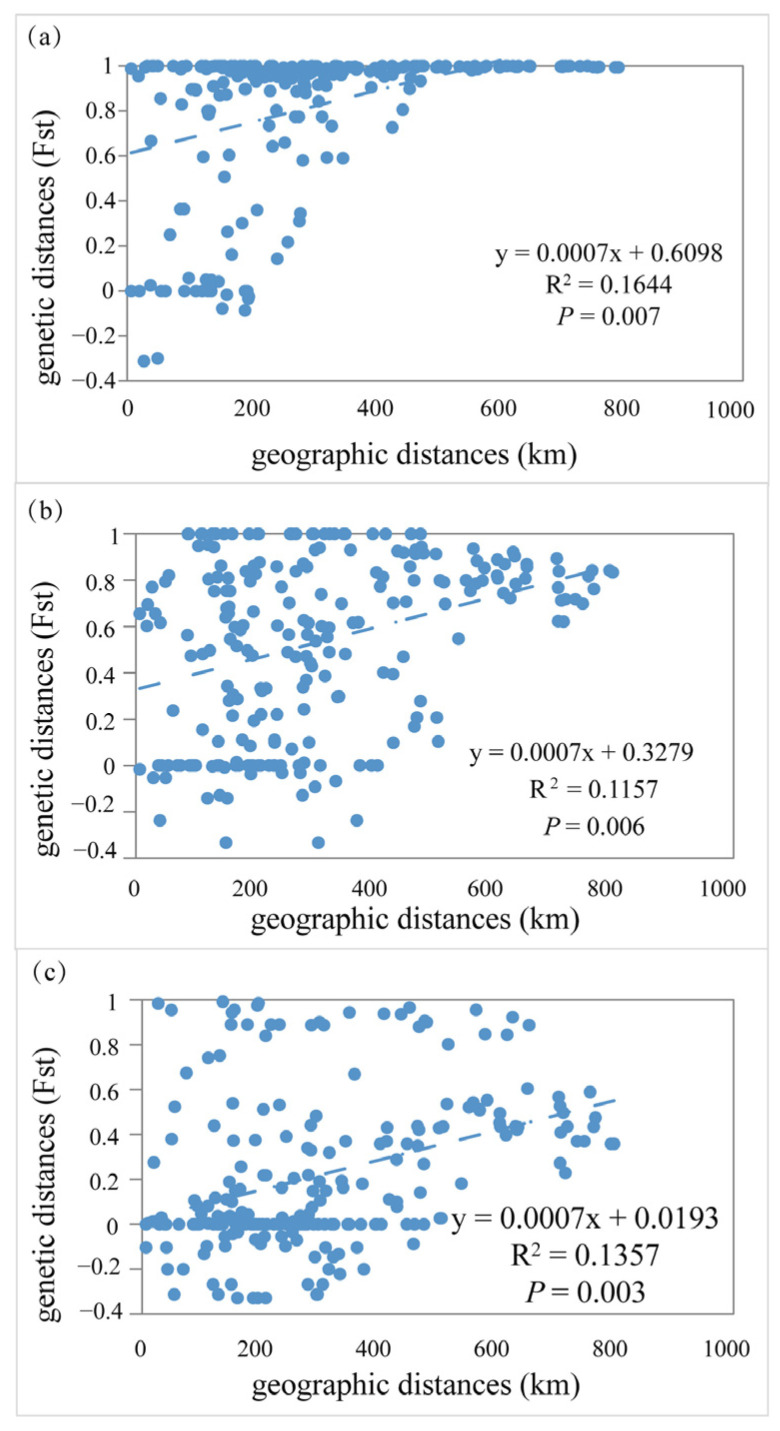
Scatter plots of genetic distance vs. geographical distance (km: kilometer) for pairwise population comparisons inferred from mtDNA (**a**), *RAG1* (**b**), and *PLAGL2* (**c**).

MtDNA-based pairwise genetic differentiation *F*st between the 22 sites ranged from −0.08 to 1.00 based on the mtDNA (Table S6). Pairwise estimates of *F*_st_ between populations ranged from −0.09 to 0.98 based on the *RAG1* and ranged from −0.33 to 0.98 based on the *PLAGL2* sequences (Tables S7 and S8). In addition, the Mantel test generated r values of 0.263 (*p* = 0.007), 0.397 (*p* = 0.006), and 0.308 (*p* = 0.028) for mtDNA, *RAG1*, and *PLAGL2* data, respectively, when evaluating the genetic diversity and geographical distance in the *P. anomala* populations (Figure 4).

### 3.4. Population Genetic Structure and Historical Population Dynamics of P. anomala

SAMOVA results showed that the highest *F*_CT_ value (*F*_CT_ = 0.835) was a grouping arrangement of Clade I and the remaining population, which geographically aligned with Clade II (Table 4). AMOVA was used to find the best phylogeographic pattern for *P. anomala* from two grouping options identified by present drainage or results of genetic analyses (Table 5). Hierarchical AMOVA revealed that grouping according to the phylogenetic tree result resulted in the highest among-group variation (*F*_CT_ = 0.793, *p* < 0.001) and was inferred to be the most probable geographical subdivision (Table 5). Finally, based on the nuclear gene *RAG1*, genetic variation within populations was the main source of total variation, with the proportion of variation within populations being 71.97%. Based on the nuclear gene *PLAGL2*, genetic variation within populations was the main source of total variation, with the proportion of variation within populations being 88.02%.

**Figure 5:**
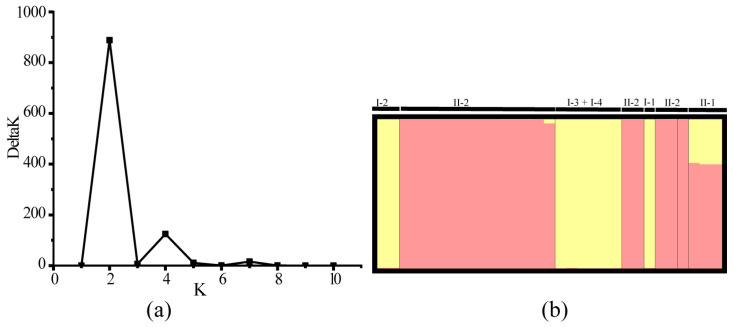
The results of structure analysis: (**a**) plot of delta K distribution of *P. anomala* sites; (**b**) histogram of the structure analysis for the model with K = 2; the *x*-axis shows clades, the proportion of each color represents the likelihood of each individual being assigned to the corresponding cluster.

The STRUCTURE analysis based on mtDNA data demonstrated that the most likely number of genetic clusters for the *P. anomala* populations was two (mean lnP(K) = –268.970; mean Delta K = 888.563) (Figure 5a). Cluster 1 included populations I-1 (Guijiang River), I-2 (Duliujiang River), and I-3 and I-4 (Yangtze River). Cluster 2 comprised populations II-1 (Nanpanjiang River) and II-2 (Hongshui River, Rongjiang River, Youjiang River, and Dahuanjiang River) (Figure 5b).

**Figure 6:**
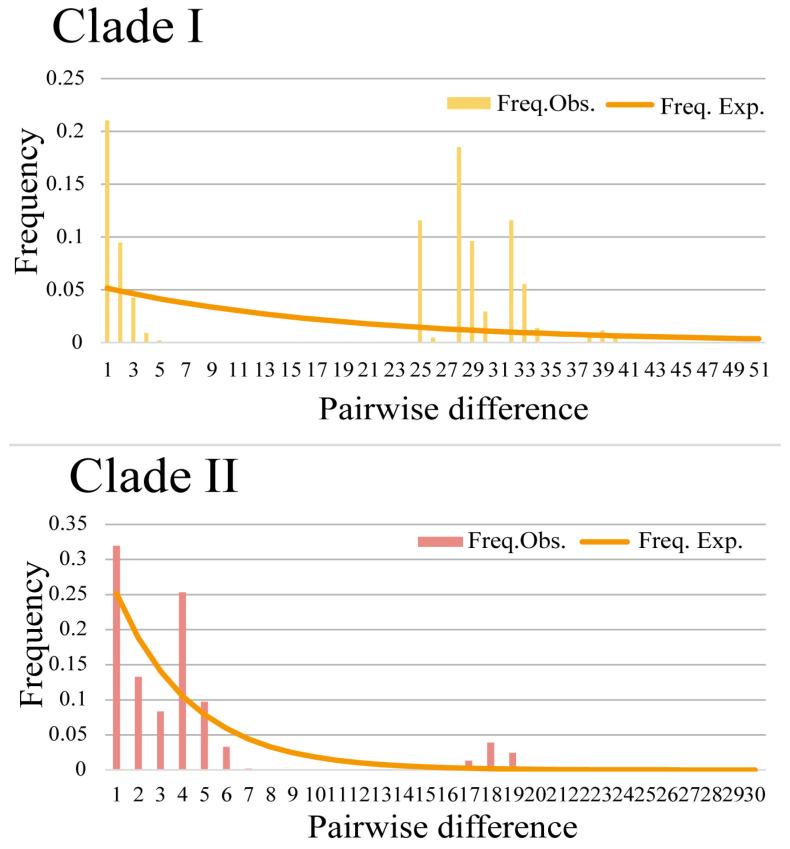
Mismatch distributions of two clades of *P. anolama* inferred from mtDNA sequences.

The historical population dynamics of *P. anomala* were analyzed using the two genetic clusters (Clade I and Clade II) identified from the AMOVA analysis. These analyses provided crucial insights into the evolutionary history of *P. anomala* (Table 6; Figure 6). Clade I exhibited a multimodal distribution with steep curves, characterized by relatively high levels of haplotype and nucleotide diversity. The positive but nonsignificant Tajima’s *D* and Fu’s *F*s values for Clade I suggested that a population expansion model could be rejected. In contrast, Clade II showed a unimodal distribution with smooth curves, indicative of population expansion. The negative and significant Tajima’s *D* and Fu’s *F*s values for Clade II further supported the inference of a historical population expansion. Additionally, the haplotype network of Clade II, centered around haplotype Hap6, displayed a star-like phylogenetic structure (Figure 3). This pattern was consistent with rapid population expansion and further corroborated the demographic inference for Clade II.

### 3.5. Estimation of Divergence Time

**Figure 7:**
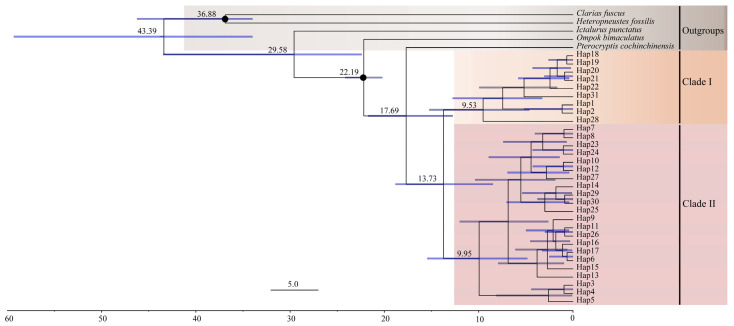
Time tree of the species *P. anomala* based on the mtDNA data. Black dots mark the calibration nodes. Numbers at nodes indicate the main divergence times of the haplotypes of the species.

Estimated divergence times based on the mtDNA data among the clades of *P. anomala* are shown in Figure 7. The divergence age estimates indicated that the split between *P. anomala* and *P. cochinchinensis* occurred around 17.69 million years ago (Mya; 95% HPD, 12.77–21.72 Mya; Figure 7). The divergence time analysis further revealed that the two main clades diverged approximately 13.73 Mya (95% HPD = 8.50–18.81 Mya; Figure 7).

## 4. Discussion

### 4.1. Genetic Diversity of P. anomala

Genetic diversity is a fundamental condition for species to maintain their evolutionary potential and is crucial for the formation, development, and sustainability of biodiversity. It enables species to adapt to complex and changing environments, thereby ensuring their survival [56,57,58]. Nevo [59] proposed that genetic diversity is correlated with environmental heterogeneity across different spatial scales, highlighting the importance of diverse habitats in maintaining genetic variation. Based on mtDNA, this study analyzed the genetic diversity of populations of *P. anomala* in Clade I and Clade II. The results showed that the genetic diversity in Clade I (h = 0.790, π = 0.01030) was higher than that in Clade II (h = 0.681, π = 0.00262). This difference may be closely related to the geographical environment in which Clade II is located. Further analysis indicated significant genetic differentiation between the population in the Hongshui River and the populations in the Guijiang and Rongjiang Rivers. This differentiation may stem from the limited dispersal ability of *P. anomala* itself, as well as the combined effects of geographical isolation and environmental differences. Compared with other fish species, the genetic diversity of Clade II is lower than that of the *Pelteobagrus fulvidraco* (Richardson, 1846) (h = 0.848, π = 0.0480) [60] and the *Ptychidio jordani* Myers, 1930 (h = 0.768, π = 0.0023) [61], which are also distributed in the Pearl River. This difference may be closely related to the ecological habits and habitat characteristics of *P. anomala*. Once a species adapts to cave-dwelling life, reduced selective pressures and smaller population sizes can exacerbate genetic drift, thereby reducing genetic diversity [62]. Moreover, genetic differentiation coefficients (*F*st) based on mitochondrial DNA, *RAG1*, and *PLAGL2* sequences, as well as Mantel tests, showed significant genetic differentiation between populations of *P. anomala* in Clade I and Clade II, revealing distinct geographical distribution patterns. These findings were also supported by phylogenetic tree and haplotype network analyses. The observed genetic differentiation may be due to the marked contrast between the tropical maritime climate of the Pearl River and the temperate monsoon climate of the Yangtze River basin. This significant north–south climatic difference may be one of the important factors leading to genetic differentiation. In addition, there were also considerable differences between the Pearl River and Yangtze River ecosystems in terms of water temperature, flow velocity, water quality, and other aspects. These factors, acting together, further exacerbate the genetic differentiation between the two populations [63].

### 4.2. Genetic Structure of P. anomala

In this study, by integrating mtDNA and nuclear DNA molecular markers of *P. anomala* populations, we systematically analyzed the population genetic structure characteristics of this species. Notably, we observed a significant incongruence between nuclear DNA haplotypes and mitochondrial gene haplotype networks. Comparative analysis indicated that the phylogeographical structure reconstructed based on mitochondrial gene markers exhibited a more pronounced phylogeographical differentiation pattern than nuclear gene data, while the phylogenetic topology constructed by nuclear genes failed to resolve distinct evolutionary clades. This phenomenon of incongruence between mitochondrial and nuclear DNA is widespread in many organisms [64,65]. Such incongruence typically implies ancient hybridization events and incomplete lineage sorting (ILS) [66]. Mitochondrial genes, due to their faster substitution rates and smaller effective population sizes, are more susceptible to hybridization in phylogenetic analyses. In contrast, nuclear genes, with their slower substitution rates and larger effective population sizes, are more prone to ILS in phylogenetic analyses [66,67]. Additionally, the relatively slow mutation rate of nuclear genes somewhat limits their ability to resolve population structure, a phenomenon that has been verified in studies of the *Baryancistrus xanthellus* Rapp Py-Daniel, Zuanon & de Oliveira, 2011 [68].

The phylogenetic trees and haplotype network based on mtDNA data revealed the genetic differentiation characteristics of *P. anomala* populations. The research findings indicated that *P. anomala* populations could be divided into two main clades, with their distribution closely related to geographical regions (Figure 2). These results suggested that the Yangtze River population shared a relatively close genetic relationship with the populations of the Guijiang and Duliujiang Rivers. Similar patterns had also been reported in *Hemibarbus medius* Yue, 1995 and *Hemibarbus labeo* (Pallas, 1776) [69], as well as *Hypophthalmichthys nobilis* (Richardson, 1845) [70]. The close genetic relationships among subclades within Clade I and the current distribution patterns of Clade I and Clade II can be attributed to the following factors.

First, the monsoon climate impacts brought about by the uplift of the QTP, as well as the formation of the Nanling Mountains, have played significant roles. Phylogenetic analysis using BEAST estimated that these lineages diverged approximately 8.5–18.81 mya (Figure 7), a period that was potentially influenced by the southwest monsoon [71]. During the Miocene, as the QTP approached its present-day elevation, its uplift significantly intensified the monsoon systems and altered marine biogeochemical processes. This process not only drove the South Asian and East Asian monsoons into an enhanced phase but also fundamentally changed the evolution of the regional topography [72,73,74,75]. The mid-Miocene climatic optimum (approximately 17–14 mya) saw intensified monsoon precipitation that dramatically accelerated karst cave system development [76]. Studies have shown that the ancestral populations of the cave-dwelling and non-cave-dwelling groups of the genus *Triplophysa* initially diverged around 15.3–13 million years ago, which is inferred to be closely related to the continuous uplift of the plateau [72]. We speculate that the differentiation of populations of the *P. anomala* inhabiting the same karst environment is also likely to be significantly associated with the ongoing uplift of the QTP. Concurrently, the formation of the Nanling Mountains as a geographical barrier played a significant role in the genetic differentiation of these fish populations. During its early uplift, the Nanling Mountains had a relatively low elevation, which did not effectively block gene flow between freshwater fish populations on either side [69]. However, during the uplift of the QTP, the Nanling Mountains experienced accelerated uplift [77]. This rapid uplift transformed the Nanling Mountains into a geographical barrier between the northern and southern river systems [69]. The continued uplift ultimately established a divide between the Pearl River and Yangtze River systems. This divide prevented warm air masses south of the mountains from moving northward and blocked cold air masses north of the mountains from penetrating southward. Consequently, a climatic boundary formed between the south subtropical and mid-subtropical zones, restricting gene flow and leading to genetic differentiation [78]. In summary, the uplift of QTP and the formation of the Nanling Mountains as a geographical barrier both contributed to the genetic differentiation observed in fish populations in the region.

Second, river capture events played a role. Tectonic movements and river capture events led to connections or diversions between the Yangtze River and Pearl River systems. Early geological and ichthyological studies showed that some rivers in the Pearl River and Yangtze River were geographically very close, with a few even being directly connected [79,80]. For example, the Lingqu Canal, which connected the Xiangjiang (a tributary of the Yangtze River) and Guijiang Rivers [5,80], may also have promoted gene flow between populations of the Yangtze River and Pearl River systems.

The results of the haplotype network analysis showed that there is haplotype sharing between the Pearl River and the Yangtze River (Figures S1 and S2). It is inferred that this phenomenon is more likely the result of the combined effects of the dispersal of the Yangtze River population and shared ancestral polymorphisms. According to the studies of Yang et al., (2022) and Li et al., (2020), if ancestral polymorphisms are shared between the Pearl River and Yangtze River, each basin should retain a subset of unique haplotypes [70,81]. However, the haplotype network reveals that, despite shared haplotypes, both basins exhibit distinct unique haplotypes. This indicates that genetic differentiation between populations is not solely attributable to ancestral polymorphism retention, and dispersal of the Yangtze River population likely contributed to the observed pattern. Secondly, the analysis of average genetic distances between populations (Table 3) showed that the genetic distance between populations of Clade II and the four subclades of Clade I increased gradually from north to south. This suggested that some ancestors of the *P. anomala* populations in the Pearl River likely originated from the Yangtze River. It was worth noting that, in the Pearl River and Yangtze River, genetic differentiation among different fish populations exhibited diverse characteristics. For example, the genetic distance among individuals of *P. anomala* ranged from 0% to 4%, a value significantly lower than that of the sympatric species *Squaliobarbus curriculus* (Richardson, 1846) (with genetic distances ranging from 0% to 7.43%) [78] but slightly higher than the genetic distance between populations of *Hypophthalmichthys molitrix* (Valenciennes, 1844) across basins (at 2.3%) [70]. Although *P. anomala* (0–4%), *H. molitrix* (2.3%), and *S. curriculus* (0–7.43%) all showed clear genetic differentiation between populations in the Pearl River and Yangtze River, this degree of differentiation had not reached the level of speciation. This demonstrated that there is no correspondence between genetic distance and species boundaries, and genetic differentiation between populations, even up to 7.43%, might still fall within the range of intraspecific variation. Therefore, when delimiting species, it is not sufficient to rely solely on genetic distance as a single indicator. Instead, multidimensional evidence, such as morphological differences, reproductive isolation, and ecological niche differentiation, should be taken into account [82].

### 4.3. Implications for Conservation

Deciphering the genetic structure of specific species can provide robust support for species management and conservation [83]. Given that *P. anomala*’s wild resources are scarce and artificial breeding has not yet been successful, refining conservation units and implementing in situ conservation is particularly crucial. Therefore, analyzing the genetic structure of *P. anomala* across different river systems through phylogeographic analysis is of great significance for accurately delineating its conservation units. In our study, we observed two clades in *P. anomala* populations, which we proposed should be recognized as two molecular operational taxonomic units (MOTUs). Although *P. anomala* is currently listed as a species of Least Concern on the IUCN Red List due to its wide distribution and seemingly stable population [84], our mitochondrial DNA (mtDNA) analysis revealed relatively low haplotype and nucleotide diversity in the Youjiang, Rongjiang, and Guijiang River basins. The genetic diversity of these populations was significantly lower than that of *Pelteobagrus fulvidraco* and *Hemibarbus medius* in the same river basins. Moreover, the analysis of genetic diversity using nuclear gene markers also showed relatively low haplotype and nucleotide diversity in the Nanpanjiang, Duliujiang, Dahuanjiang, and Hongshui Rivers. The genetic diversity in Duliujiang and Dahuanjiang Rivers might have been limited by the small sample size. Cave-dwelling fish, due to their unique habitats, face multiple threats including habitat degradation, environmental pollution, overexploitation of resources, and invasion of non-native species. In China, most cave-dwelling fish have limited distribution ranges and small population sizes, making them highly vulnerable to even minor threats [85]. To protect these species, effective conservation measures must be implemented, including strengthening habitat protection, reducing pollution sources, managing resource exploitation sustainably, and preventing the invasion of non-native species, to ensure their survival and reproduction.

## 5. Conclusions

This study has conducted a focused and comprehensive investigation into the phylogeography and diversity of *P. anomala* in Southwest China. The identification of two distinct genetic lineages highlights the pivotal role of geological events and river systems in shaping the evolutionary trajectory of the species. Influenced by geographical and environmental factors, the relatively low genetic diversity and high genetic differentiation among evolutionary branches underscore the necessity of considering fine-scale spatial heterogeneity when devising conservation strategies. In summary, this study emphasizes the critical impact of geological, climatic, and riverine factors on the distribution and evolutionary history of *P. anomala* in the Yangtze and Pearl River and highlights the urgency of further research to protect this species. We plan to expand the scope of our study and increase the sample size in future research and combine more types of markers (such as SNP, SSR, etc.) to comprehensively assess the population genetic structure of *P. anomala*, thereby providing a more scientific and systematic basis for the formulation of its conservation strategies.

## Figures and Tables

**Table 1:** Basic information for sampling sites.

Population Code	Drainage System	Sampling Site	Longitude (°E)	Latitude (°N)	Sample Size
N1	Nanpanjiang River	Gaoka Village, Guizhou	104.8581	25.0310	2
N2	Nanpanjiang River	Louxia Town, Guizhou	104.8766	25.3446	2
N3	Nanpanjiang River	Haiziba Village, Guangxi	105.1925	24.8566	3
N4	Nanpanjiang River	Zuoshe Village, Guizhou	105.1196	25.2569	9
N5	Nanpanjiang River	Kewang Village, Guizhou	105.2134	25.1208	5
H1	Hongshui River	Aozizhai Village, Guizhou	105.6380	26.0297	22
H2	Hongshui River	Dongtang Village, Guangxi	107.5182	25.1513	7
H3	Hongshui River	Dalongguan Village, Guangxi	106.4319	24.5694	6
H4	Hongshui River	Bali Village, Guangxi	106.4669	24.5272	6
H5	Hongshui River	Dajing Village, Guizhou	106.8625	25.5571	16
H6	Hongshui River	Ganlongdong Village, Guizhou	105.6773	25.2779	24
H7	Hongshui River	Jiazuan Town, Guangxi	107.1531	24.1990	15
H8	Hongshui River	Shanglin County, Guangxi	108.6050	23.4324	5
H9	Hongshui River	Fengting Village, Guizhou	106.5623	25.4134	17
H10	Hongshui River	Bina Village, Guizhou	106.7782	25.7632	2
DH	Dahuanjiang River	Jialiang Town, Guizhou	107.9958	25.2354	5
DL	Duliujiang River	Bakai Town, Guizhou	108.3740	25.8536	2
RJ	Rongjiang River	Rongan County, Guangxi	109.3975	25.2245	10
JX	Youjiang River	Longteng Village, Guangxi	106.3436	23.0566	16
GJ	Guijiang River	Dalingshan Village, Guangxi	110.7352	24.9354	15
Y1	Yangtze River	Heboling Village, Hunan	111.9863	25.7043	40
Y2	Yangtze River	Guping Village, Hunan	111.2851	26.7840	24

**Table 2:** Summary of genetic diversity based on mtDNA, *RAG1*, and *PLAGL2* data in *P. anomala* in Southwest China.

MtDNA					*RAG1*				*PLAGL2*			
River System	N	nh	π	h	N	nh	π	h	N	nh	π	h
Nanpanjiang River	21	4	0.00641 ± 0.0011	0.533 ± 0.111	22	8	0.00451 ± 0.0003	0.665 ± 0.050	22	7	0.03086 ± 0.0158	0.481 ± 0.131
Hongshui River	120	15	0.00137 ± 0.0002	0.536 ± 0.052	93	5	0.00060 ± 0.0001	0.361 ± 0.036	112	8	0.00081 ± 0.0003	0.122 ± 0.042
Yangtze River	64	8	0.00750 ± 0.0005	0.714 ± 0.032	52	6	0.00103 ± 0.0002	0.558 ± 0.023	67	13	0.00140 ± 0.0004	0.420 ± 0.077
Duliujiang River	2	2	0.00056 ± 0.0003	1.000 ± 0.500	1	1	0	0	2	1	0	0
Dahuanjiang River	5	3	0.00056 ± 0.0002	0.700 ± 0.218	4	1	0	0	2	1	0	0
Youjiang River	16	2	0.00007 ± 0.0001	0.125 ± 0.106	26	9	0.00365 ± 0.0004	0.590 ± 0.063	20	11	0.05209 ± 0.0204	0.874 ± 0.064
Rongjiang River	10	1	0	0	19	13	0.00603 ± 0.0005	0.845 ± 0.039	15	8	0.00433 ± 0.0038	0.838 ± 0.085
Guijiang River	15	1	0	0	12	2	0.00020 ± 0.0002	0.083 ± 0.075	15	2	0.00020 ± 0.0002	0.133 ± 0.112

The number of haplotypes (nh), nucleotide diversity (π), and haplotypes diversity (h).

**Table 3:** The Kimura 2-parameter (K2P) distances (%) of *P. anomala* populations inferred from mtDNA data.

	I-1	I-2	I-3	I-4	II-1	II-2
I-1						
I-2	0.014					
I-3	0.014	0.016				
I-4	0.018	0.022	0.016			
II-1	0.031	0.034	0.033	0.038		
II-2	0.032	0.036	0.034	0.040	0.006	

**Table 4:** SAMOVA results based on mtDNA (K = 2) of 22 *P. anomala* sites from Southwest China.

K	Source of Variation	d.f.	Sum of Squares	Variance Components	Percentage of Variation	*p* Value
2	Among clades	1	2070.317	18.03249	83.53	0.000
	Among populations within regions	20	711.765	3.39220	15.71	0.000
	Within populations	231	37.495	0.16231	0.75	0.000
	Total	252	2819.577	21.58701		

**Table 5:** Analysis of molecular variance (AMOVA) of *P. anomala* based on mtDNA data. Significance test: 1000 permutations.

Grouping Options	Variation (%)			*F*_CT_	*F*_SC_	*F*_ST_
	Among Clades	Among Populations Within Clades	Within Populations			
1. The Pearl River and the Yangtze River						
	62.11	29.50	8.38	0.621 *	0.779 ***	0.916 ***
2. Phylogenetic tree groupings						
	79.27	12.50	8.23	0.793 *	0.603 ***	0.918 ***

Note: *: *p* < 0.05; ***: *p* < 0.001.

**Table 6:** Statistics of neutrality test and mismatch distribution analysis results for two lineages of *P. anomala*.

Grouping (Populations)	π	h	Tajima’s *D* (*p* Value)	Fu’s *F*s (*p* Value)
Clade I	0.01030	0.790	1.37841 (0.936)	15.00847 (0.995)
Clade II	0.00262	0.681	−1.49888 (0.040)	−5.68864 (0.052)

## Data Availability

All sequences generated during this study have been deposited in GenBank (Accession numbers: PV411066−PV411294, PV411932−PV412184, PV540427−PV540681, PV546889−PV547139).

## Supplementary Materials

The following supporting information can be downloaded at: https://www.mdpi.com/article/10.3390/ani15091202/s1, Figure S1: The distribution of the haplotypes based on *RAG1* in the *P. anomala* populations; each haplotype is represented by a circle. The size of the circle is proportional to that haplotype’s frequency. Different colors indicate different clades; Figure S2: The distribution of the haplotypes based on *PLAGL2* in the *P. anomala* populations; each haplotype is represented by a circle. The size of the circle is proportional to that haplotype’s frequency. Different colors indicate different clades; Table S1: Primers information; Table S2: Geographic distance (km) among populations of *P. anomala;* Table S3: The distribution of the haplotypes based on mtDNA in the *P. anomala* populations; Table S4: The distribution of the haplotypes based on *RAG1* in the *P. anomala* populations; Table S5: The distribution of the haplotypes based on *PLAGL2* in the *P. anomala* populations; Table S6: Pairwise genetic differentiation of *P. anomala* populations inferred from mtDNA; Table S7: Pairwise genetic differentiation of *P. anomala* populations inferred from *RAG1;* Table S8: Pairwise genetic differentiation of *P. anomala* populations inferred from *PLAGL2;* Table S9: Analysis of molecular variance (AMOVA) of *P. anomala* based on *RAG1* and *PLAGL2* data. Significance test: 1000 permutations.

## Abbreviations

The following abbreviations are used in this manuscript:

QTP	Qinghai–Tibet Plateau
Mya	Million years ago
ILS	Incomplete lineage sorting
