# Supplementary material for: Genetic Structure of the Cave-Dwelling Catfish Pterocryptis anomala (Siluriformes: Siluridae) in Southwest China
Source: Animals (Basel). 2025 Apr 23;15(9):1202. doi: 10.3390/ani15091202 (PMC12070860; doi:10.3390/ani15091202)
Supplement: Supplementary file 1 [file animals-15-01202-s001.zip › animals-3545799-supplementary.pdf]

Supplementary Materials

Table S1. Primers information.

Gene	Primer code	Primer sequence (5′-3′)	Annealing temperature (℃)	PCR steps	Product length (bp)	Reference
COI	COI-F	TCAACCAACCACAAAGACATTGGCAC	55	PCR	651	Ward et al., 2005
	COI-R	TAGACTTCTGGGTGGCCAAAGAATCA				
Cyt *b*	L14724	GACTTGAAAAACCACCGTTG	55	PCR	1138	Xiao et al., 2001
	H15915	CTCCGATCTCCGGATTACAAGAC				
PLAGL2	Plagl2_F9	CCACACACTCYCCACAGAA	55	1 st PCR	660	Li et al., 2007
	Plagl2_R930	TTCTCAAGCAGGTATGAGGTAGA				
	Plagl2_F51	AAAAGATGTTTCACCGMAAAGA		2 nd PCR		
	Plagl2_R920	GGTATGAGGTAGATCCSAGCTG				
RAG1	RAG1_L3a	GCRTTNCCAATGTCACARTG	57	1 st PCR	766	Cramer et al., 2011
	RAG1_MFL1	AGCTGCAGYCARTAYCAYAARATGTA				
	RAG1_CCF	TGGACGTCGATCTTTCAACCC	55	2 nd PCR		
	RAG1_CCR	CTAATGTGGGCTGTGTCTCCA				

References

Ward, R.; Zemlak, T.; Innes, B.; Last, P.; Hebert, P. DNA Barcoding Australia's fish species. *PHILOS. T. R. SOC. B*. **2005,** 360. 1847-1857. https://doi.org/10.1098/rstb.2005.1716.

Xiao, W.H.; Zhang, Y.P.; Liu, H.Z. Molecular systematics of Xenocyprinae (Teleostei: Cyprinidae): Taxonomy, biogeography, and coevolution of a special group restricted in East Asia. *Mol. Phylogenet. Evol.* **2001,** 18, 163–173. https://doi.org/10.1006/mpev.2000.0879.

Li, C.H.; Ortí, G.; Zhang, G.; Lu, G.Q. A practical approach to phylogenomics: the phylogeny of ray-finnedfish (Actinopterygii) as a case study. *BMC Evol. Biol*. **2007,** 7, 44. https://doi.org/10.1186/1471-2148-7-44.

Cramer, C.A.; Bonatto, S.L.; Reis, R.E. Molecular phylogeny of the Neoplecostominae and Hypoptopomatinae (Siluriformes: Loricariidae) using multiple genes. *Mol. Phylogenet. Evol.* **2011,** 59, 43–52. https://doi.org/10.1016/j.ympev.2011.01.002

Table S2. Geographic distance (km) among *P. anomala* populations

	DL	N1	N2	H1	H2	H3	H4	DH	H5	H6	N3	H7	H8	H9	N4	Y2	RJ	GJ	YJ	N5	H10	Y1
**DL**																						
**N1**	404.3																					
**N2**	394.3	38.6																				
**H1**	305.3	150.7	119.7																			
**H2**	128.7	296.4	295.0	235.7																		
**H3**	267.7	184.1	197.5	200.3	140.4																	
**H4**	267.7	189.4	203.4	206.7	139.8	6.4																
**DH**	87.1	350.1	347.4	280.1	54.1	192.3	191.0															
**H5**	172.2	232.3	222.6	148.3	88.4	130.5	133.9	132.2														
**H6**	308.4	96.1	89.5	92.9	205.5	120.9	127.3	258.1	136.3													
**N3**	374.8	42.9	69.5	152.8	261.4	142.3	147.4	315.5	204.9	74.7												
**H7**	244.5	275.0	289.7	281.2	123.5	92.1	86.2	157.9	169.7	210.8	232.1											
**H8**	297.6	460.5	476.3	458.7	242.3	278.7	272.8	230.4	324.3	396.3	417.7	186.6										
**H9**	208.8	195.4	187.8	128.0	111.1	104.6	109.3	161.0	37.8	99.9	167.1	162.8	332.2									
**N4**	369.6	40.2	29.1	111.5	267.3	168.7	174.6	320.1	197.5	62.1	49.8	260.8	447.4	161.7								
**Y2**	402.5	797.8	792.6	707.8	502.0	633.8	631.2	447.9	570.6	704.2	763.4	568.8	467.9	604.8	766.3							
**RJ**	137.8	505.8	503.4	430.1	209.3	339.7	337.2	156.0	285.1	414.1	470.2	279.6	236.0	316.4	476.2	294.1						
**GJ**	286.1	654.3	654.1	583.2	359.0	481.1	477.7	307.1	437.8	564.6	617.0	408.8	299.7	468.2	626.3	168.4	153.0					
**YJ**	292.0	292.0	323.5	372.2	286.9	18.3	179.4	323.2	310.8	281.0	253.9	165.5	255.8	288.7	301.0	706.6	430.7	539.8				
**N5**	363.2	41.0	6.5	121.7	256.5	151.5	157.3	310.0	191.2	55.1	32.5	243.6	430.5	154.4	19.7	757.3	465.9	615.1	281.4			
**H10**	177.9	232.0	217.9	131.1	111.7	151.8	155.9	150.3	27.1	136.4	208.8	196.6	350.4	49.4	194.9	579.7	299.7	452.1	334.4	191.3		
**Y1**	344.0	747.6	735.3	635.5	465.5	605.5	604.4	413.3	515.3	651.5	718.8	559.6	508.7	552.5	711.9	155.0	285.1	236.7	715.5	706.5	517.4	

Table S3. The distribution of the haplotypes based on mtDNA in the *P. anomala* populations.

Site / Haplotype number	Duliujiang River	Nanpanjiang River	Hongshui River	Dahuanjiang River	Rongjiang River	Yangtze River	Guijiang River	Youjiang River	Total
1	1								1
2	1								1
3		2							2
4		4	1						5
5		1							1
6		14	80						94
7			3						3
8			5						5
9			1						1
10			17						17
11			1						1
12			1						1
13			1						1
14			4						4
15			2						2
16			1						1
17			1						1
18						35			35
19						2			2
20						1			1
21						1			1
22						1			1
23				3					3
24				2					2
25			1		10				11
26			1						1
27			1						1
28							15		15
29								15	15
30								1	1
31						24			24

Table S4. The distribution of the haplotypes based on *RAG1* in the *P. anomala* populations.

Site / Haplotype number	Duliujiang River	Nanpanjiang River	Hongshui River	Dahuanjiang River	Rongjiang River	Yangtze River	Guijiang River	Youjiang River	Total
1	2	21	144		4			13	184
2			1						1
3			38	8	11	2			59
4			1						1
5		2							2
6		1				53	23	1	78
7						1			1
8					2				2
9		15	2		10			31	58
10					1				1
11					2				2
12		1			1			1	3
13					1				1
14					1				1
15					2				2
16					1				1
17					1				1
18					1				1
19								2	2
20								1	1
21								1	1
22								1	1
23								1	1
24		1							1
25		1							1
26		2							2
27							1		1
28						45			45
29						2			2
30						1			1

Table S5. The distribution of the haplotypes based on *PLAGL2* in the *P. anomala* populations.

Site<br>Haplotype number	Duliujiang River	Nanpanjiang River	Hongshui River	Dahuanjiang River	Rongjiang River	Yangtze River	Guijiang River	Youjiang River	Total
1	2	16	105	2	6	4		2	137
2			1						1
3			1						1
4			1						1
5			1						1
6			1						1
7						51	14		65
8						1			1
9					1	2			3
10						1			1
11						1			1
12						1			1
13						1			1
14			1		2			2	5
15			1		2			7	10
16					1			2	3
17					1				1
18					1				1
19					1				1
20							1		1
21		1							1
22		1						1	2
23								1	1
24								1	1
25								1	1
26								1	1
27								1	1
28								1	1
29		1							1
30		1							1
31		1							1
32		1							1
33						1			1
34						1			1
35						1			1
36						1			1

37	1	1

Table S6. Pairwise genetic differentiation of *P. anomala* populations inferred from mtDNA.

	DL	N1	N2	H1	H2	H3	H4	DH	H5	H6	N3	H7	H8	H9	N4	Y2	RJ	GJ	YJ	N5	H10	Y1
DL																						
N1	0.98																					
N2	0.97	0.66																				
H1	0.99	1.00	0.99																			
H2	0.98	0.97	0.96	0.97																		
H3	0.99	1.00	0.99	1.00	0.91																	
H4	0.99	1.00	0.99	1.00	0.91	0.00																
DH	0.98	0.97	0.96	0.97	0.85	0.89	0.89															
H5	0.98	0.95	0.95	0.04	0.82	0.80	0.80	0.78														
H6	0.99	1.00	0.99	0.00	0.97	1.00	1.00	0.97	0.04													
N3	0.99	1.00	0.25	1.00	0.97	1.00	1.00	0.97	0.95	1.00												
H7	0.95	0.88	0.88	0.34	0.59	0.36	0.36	0.50	0.16	0.36	0.88											
H8	0.97	0.94	0.93	0.89	0.80	0.77	0.77	0.73	0.59	0.90	0.95	0.30										
H9	0.98	0.97	0.97	0.05	0.89	0.89	0.89	0.87	0.02	0.05	0.97	0.26	0.73									
N4	0.99	1.00	0.99	0.00	0.94	1.00	1.00	0.94	-0.02	0.00	1.00	0.21	0.80	-0.01								
Y2	0.99	0.99	0.99	0.99	0.99	0.99	0.99	0.99	0.99	0.99	0.99	0.98	0.99	0.99	0.99							
RJ	0.99	1.00	0.99	1.00	0.93	1.00	1.00	0.92	0.58	1.00	1.00	0.31	0.64	0.77	1.00	0.99						
GJ	0.99	1.00	0.99	1.00	0.99	1.00	1.00	0.99	0.98	1.00	1.00	0.97	0.99	0.90	1.00	0.99	1.00					
YJ	0.99	0.99	0.98	0.98	0.92	0.95	0.95	0.91	0.84	0.98	0.99	0.60	0.65	0.91	0.97	0.99	0.96	0.99				
N5	0.99	1.00	0.98	0.00	0.92	1.00	1.00	0.91	-0.08	0.00	1.00	0.14	0.72	-0.07	0.00	0.99	1.00	1.00	0.96			
H10	0.98	1.00	0.97	0.00	0.89	1.00	1.00	0.86	-0.31	0.00	1.00	-0.03	0.59	-0.30	0.00	0.99	1.00	1.00	0.96	0.00		
Y1	0.99	1.00	0.99	1.00	0.99	1.00	1.00	0.99	0.99	1.00	1.00	0.98	0.99	0.99	1.00	0.99	1.00	1.00	0.99	1.00	1.00	

Table S7. Pairwise genetic differentiation of *P. anomala* populations inferred from *RAG1*.

	**DL**	**N1**	**N2**	**H1**	**H2**	**H3**	**H4**	**DH**	**H5**	**H6**	**N3**	**H7**	**H8**	**H9**	**N4**	**Y2**	**RJ**	**GJ**	**YJ**	**N5**	**H10**
DL																					
N1	0.00																				
N2	0.00	0.00																			
H1	-0.33	-0.33	-0.14																		
H2	1.00	1.00	1.00	0.85																	
H3	0.83	0.83	0.86	0.82	-0.12																
H4	1.00	1.00	1.00	0.87	0.00	-0.01															
DH	1.00	1.00	1.00	0.87	0.00	-0.03	0.00														
H5	0.00	0.00	0.00	-0.00	1.00	0.94	1.00	1.00													
H6	0.00	0.00	0.00	0.00	1.00	0.95	1.00	1.00	0.00												
N3	0.00	0.00	0.00	-0.14	1.00	0.86	1.00	1.00	0.00	0.00											
H7	-0.03	-0.03	0.10	0.24	0.49	0.47	0.56	0.54	0.28	0.32	0.10										
H8	1.00	1.00	1.00	0.85	0.00	-0.12	0.00	0.00	1.00	1.00	1.00	0.49									
H9	0.00	0.00	0.00	-0.00	1.00	0.94	1.00	1.00	0.00	0.00	0.00	0.30	1.00								
N4	-0.23	-0.23	-0.05	0.15	0.46	0.51	0.58	0.55	0.19	0.23	-0.05	0.07	0.46	0.21							
Y2	0.83	0.83	0.80	0.83	0.91	0.90	0.92	0.91	0.88	0.89	0.84	0.78	0.91	0.88	0.76						
RJ	0.10	0.10	0.20	0.39	0.22	0.29	0.29	0.27	0.37	0.40	0.20	0.33	0.22	0.38	0.27	0.42					
GJ	0.85	0.85	0.86	0.85	0.93	0.91	0.94	0.94	0.92	0.93	0.86	0.77	0.93	0.93	0.72	0.01	0.34				
YJ	0.44	0.44	0.49	0.61	0.56	0.60	0.60	0.59	0.60	0.62	0.49	0.59	0.56	0.61	0.53	0.62	0.09	0.54			
N5	0.61	0.61	0.66	0.80	0.70	0.75	0.75	0.73	0.79	0.82	0.65	0.77	0.70	0.80	0.69	0.81	0.16	0.74	0.01		
H10	0.11	0.11	0.33	0.75	0.48	0.68	0.68	0.64	0.77	0.81	0.33	0.66	0.48	0.79	0.47	0.79	-0.09	0.70	-0.06	0.08	
Y1	0.69	0.69	0.72	0.78	0.80	0.80	0.82	0.81	0.79	0.80	0.71	0.75	0.80	0.80	0.71	0.65	0.47	0.60	0.62	0.76	0.69

Table S8. Pairwise genetic differentiation of *P. anomala* populations inferred from *PLAGL2*.

	**DL**	**N1**	**N2**	**H1**	**H2**	**H3**	**H4**	**DH**	**H5**	**H6**	**N3**	**H7**	**H8**	**H9**	**N4**	**Y2**	**RJ**	**GJ**	**YJ**	**N5**	**H10**
DL																					
N1	0.00																				
N2	0.00	0.00																			
H1	-0.26	-0.26	-0.26																		
H2	-0.31	-0.31	-0.31	-0.05																	
H3	0.00	0.00	0.00	-0.08	-0.09																
H4	0.00	0.00	0.00	-0.05	-0.05	0.00															
DH	0.00	0.00	0.00	-0.26	-0.31	0.00	0.00														
H5	0.00	0.00	0.00	0.04	0.11	0.00	0.00	0.00													
H6	0.00	0.00	0.00	0.08	0.22	0.00	0.00	0.00	0.00												
N3	-0.20	-0.20	-0.20	-0.04	-0.07	0.11	0.19	-0.20	0.51	0.67											
H7	0.00	0.00	0.00	0.04	0.12	0.00	0.00	0.00	0.00	0.00	0.53										
H8	0.00	0.00	0.00	-0.00	-0.10	0.00	0.00	0.00	0.00	0.00	0.11	0.00									
H9	-0.33	-0.33	-0.33	0.04	0.08	-0.13	-0.10	-0.33	0.00	0.05	0.26	0.01	-0.13								
N4	0.00	0.00	0.00	0.00	0.04	0.00	0.00	0.00	0.00	0.00	0.38	0.00	0.00	-0.04							
Y2	0.36	0.36	0.36	0.41	0.43	0.42	0.44	0.36	0.51	0.57	0.43	0.51	0.42	0.49	0.40						
RJ	0.03	0.03	0.03	0.29	0.22	0.16	0.19	0.03	0.33	0.43	0.14	0.34	0.16	0.32	0.27	0.48					
GJ	0.89	0.89	0.89	0.55	0.67	0.90	0.91	0.89	0.94	0.95	0.84	0.94	0.90	0.88	0.92	0.05	0.54				
YJ	-0.14	-0.14	-0.14	0.18	0.08	0.012	0.04	-0.15	0.15	0.22	-0.03	0.16	0.01	0.15	0.11	0.27	0.08	0.18			
N5	-0.10	-0.10	-0.10	0.44	0.21	0.10	0.15	-0.10	0.38	0.52	0.03	0.39	0.10	0.37	0.28	0.59	0.35	0.39	0.04		
H10	0.89	0.89	0.89	0.75	0.74	0.94	0.95	0.89	0.98	0.99	0.84	0.98	0.94	0.95	0.97	0.85	0.19	0.97	-0.22	-0.07	
Y1	0.37	0.37	0.37	0.43	0.44	0.44	0.45	0.37	0.54	0.60	0.44	0.54	0.44	0.52	0.49	0.02	0.44	0.03	0.23	0.53	0.80

Table S9. Analysis of molecular variance (AMOVA) of *P. anomala* based on *RAG1* and *PLAGL2* data. Significance test: 1000 permutations.

	Source of Variation	Sum of squares	Variance components	Percentage of variation
RAG1	Among clades	86.138	0.45631	28.02518
	Within populations	534.393	1.17191	71.97482
PLAGL2	Among clades	45.496	0.37912	11.98108
	Within populations	704.649	2.78517	88.01892

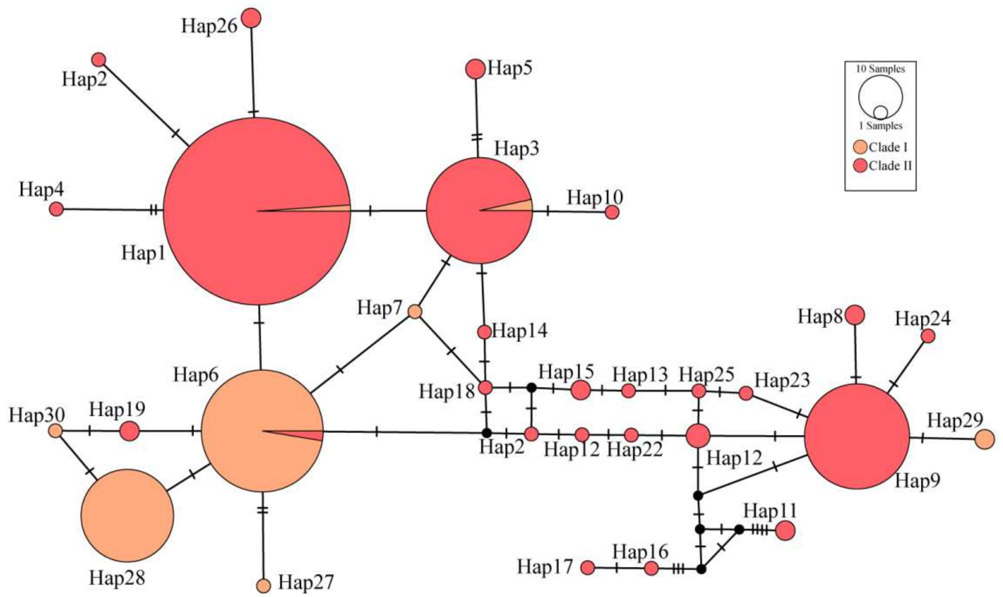

Figure S1. The distribution of the haplotypes based on *RAG1* in the *P. anomala* populations, each haplotype is represented by a circle. The size of the circle is proportional to that haplotype's frequency. Different colors do indicating different clades.

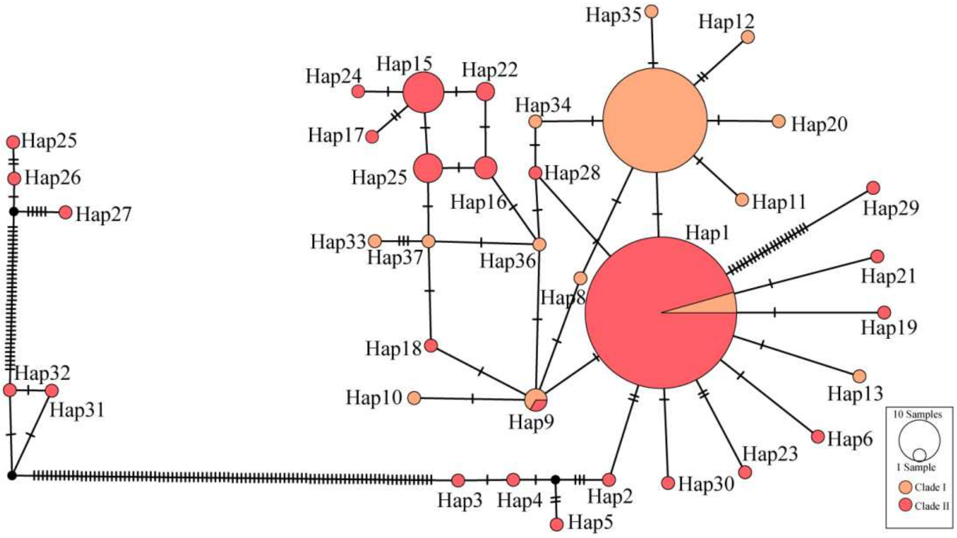

Figure S2. The distribution of the haplotypes based on *PLAGL2* in the *P. anomala* populations, each haplotype is represented by a circle. The size of the circle is proportional to that haplotype's frequency. Different colors do indicating different clades.
